# Heterozygous loss of *Engrailed-1* and α-synucleinopathy (En1/SYN): A dual-hit preclinical mouse model of Parkinson’s disease, analyzed with artificial intelligence

**DOI:** 10.1016/j.nbd.2024.106647

**Published:** 2024-08-24

**Authors:** Lucas Stetzik, Gabriela Mercado, Jennifer A. Steiner, Allison Lindquist, Carla Gilliland, Emily Schulz, Lindsay Meyerdirk, Lindsey Smith, Jeremy Molina, Darren J. Moore

**Affiliations:** a Department of Neurodegenerative Science, Van Andel Institute, Grand Rapids, MI, United States; b Aiforia Inc, Cambridge Innovation Center, Cambridge, MA, United States; c Rush University Medical Center, Chicago, IL, United States

**Keywords:** AI, Preclinical, Alpha synuclein, engrailed1, Behavior

## Abstract

In this study, we develop and validate a new Parkinson’s disease (PD) mouse model that can be used to better understand how the disease progresses and to test the effects of new, potentially disease-modifying, PD therapies. Our central hypothesis is that mitochondrial dysfunction intercalates with misfolded α-synuclein (α-syn) accumulation in a vicious cycle, leading to the loss of nigral neurons. Our hypothesis builds on the concept that PD involves multiple molecular insults, including mitochondrial dysfunction and aberrant α-syn handling. We predicted that mitochondrial deficits, due to heterozygous loss of *Engrailed-1* (*En1*+/−), combined with bilateral injections of pathogenic α-syn fibrils (PFFs), will act to generate a highly relevant PD model – the En1/SYN model. Here, *En1*+/− mice received bilateral intrastriatal stereotaxic injections of either PBS or α-syn fibrils and were analyzed using automated behavioral tests and deep learning-assisted histological analysis at 2, 4, and 6 months post-injection. We observed significant and progressive Lewy body-like inclusion pathology in the amygdala, motor cortex, and cingulate cortex, as well as the loss of tyrosine hydroxylase-positive (TH+) cells in the substantia nigra. The En1/SYN model also exhibited significant motor impairments at 6 months post-injection, which were however not exacerbated as we had expected. Still, this model has a comprehensive number of PD-like phenotypes and is therefore superior when compared to the α-syn PFF or En1+/− models alone.

## 1. Introduction

Parkinson’s disease (PD) is the second most common neurodegenerative disease with annual societal costs exceeding $52 billion in the US alone. A major unmet need for individuals affected by PD is the lack of a therapy that slows its progression (Olanow et al., 2008; Schapira, 2009; Sherer et al., 2012). Although motor deficits of PD can be successfully treated with dopaminergic drugs for several years, the effectiveness of the therapies wanes as PD progresses. The development of such therapy has been partially hindered by the lack of adequate animal models that can predict successful interventions (Langston et al., 2015; Meissner et al., 2011; Stocchi and Olanow, 2013).

Upon autopsy, PD brains display both widespread intraneuronal α-synuclein (α-syn) inclusions and neurodegeneration that is particularly pronounced in the substantia nigra (SN). Recreating these features in a single mouse PD model has proven challenging (Beal, 2010; Blesa and Przedborski, 2014). Several neuroprotective strategies developed in toxin-based or genetic models have failed in trials with PD subjects, likely because neurotoxins are effective in killing SN dopamine (DA) neurons but fail to cause protracted neurodegeneration and α-syn aggregation. Genetic models, on the other hand, generally do not display extensive nigral neuron death (Blesa and Przedborski, 2014).

When creating experimental paradigms that more accurately model PD pathology, the field typically focuses on two considerations. First, motor and non-motor symptoms in PD indicate that neuropathology in both non-DA pathways and the mesotelencephalic DA systems are important. Second, multiple cellular functions are involved in the pathogenesis of both sporadic and familial PD. Two primary candidates of these aberrant functions are mitochondrial dysfunction and handling of misfolded proteins including α-syn.

The *En1*+/− mouse displays progressive degeneration of nigral DA neurons (Nordström et al., 2015; Sonnier et al., 2007) and a specific decrease in mitochondrial complex activity (Alvarez-Fischer et al., 2011), similar to idiopathic PD (Mizuno et al., 1989; Schapira et al., 1990). This striking phenotype is caused by the deletion of one of the *En1* alleles in an *En2* wild-type context and leads to motor and non-motor deficits. However, the *En1*+/− mouse does not develop a progressive α-synucleinopathy. We therefore utilize bilateral intrastriatal injections of α-syn preformed fibrils (PFFs) to seed the aggregation of endogenous synuclein in *En1*+/− mice. It is this combination of features that define the En1/SYN model. Post-injection of PFFs, the En1/SYN model displays reproducible and robust accumulation of α-syn phosphorylated at serine-129 (pSer129- α-syn), in a manner consistent with progressive Lewy pathology (Fujiwara et al., 2002). The α-syn aggregates first develop in the SN and other brain regions innervating the striatum, and then gradually reach several additional brain structures, implying transneuronal propagation of the pathology. Thus, validation of the En1/SYN model requires a significant progressive increase of Lewy body-like inclusions in regions other than the striatum and SN.

In this study, we have developed the EN1/SYN preclinical mouse model to define the nuanced, time-dependent evolution of α-synuclein (α-syn) pathology and its systemic impacts, notably beyond the confines of the striatonigral circuit. Central to our approach is the analysis of two injection timepoints with different aims: the examination following injections at 8 weeks of age (Chatterjee et al., 2019), observed at the 6-month benchmark, aims to determine the overarching, progressive trajectory of neurodegeneration, revealing its consequential effects on both neuronal survival and motor functionality. In parallel, the assessment of specimens injected at 4 weeks of age (Nordström et al., 2015), with subsequent evaluations at 2 and 4 months, offers a granular view into the onset and initial propagation of α-syn pathology - shedding light on its immediate ramifications on neural integrity and motor dynamics. This analysis framework helps to confirm the progression of α-syn pathology, from its inception through to its mature stages, thereby improving the model’s capacity to replicate key facets of PD progression. Importantly, this model emerges as an invaluable tool not only for its fidelity in simulating the disease’s hallmarks but also for its utility in facilitating the evaluation of potential therapeutic interventions aimed at modulating the trajectory of PD.

## 2. Methods

### 2.1. Animals

All animals were bred in the vivarium at the Van Andel Institute. *En1*+/− mice intended for breeding were reconstituted from in-house frozen stocks of *En1*+/− mouse ova and sperm using SwissOF1 mice as a background strain (Sonnier et al., 2007; Sztein et al., 2000). Tissue samples from reconstituted mice were genotyped at 2 weeks of age by Transnetyx (Cordova, TN). At 6–8 weeks of age, mice were housed with age-matched conspecific Swiss OF1 mice for breeding (Charles River Labs, France). Breeding was maintained with 3–8 mating pairs to ensure genetic diversity, and litters were pseudo-counterbalanced across treatments and age groups to minimize cage effects. All mice were housed at a maximum of 4 mice per cage under a 12-h light/dark cycle with ad libitum access to water, food, and nesting material. The housing of the animals and all procedures were carried out in accordance with the Guide for the Care and Use of Laboratory Animals (United States National Institutes of Health) and were approved by the Van Andel Institute’s Institutional Animal Care and Use Committee.

### 2.2. Preformed fibril seeding α-synuclein aggregation

Mouse α-syn pre-formed fibrils (PFFs) were produced in-house, as previously described (Becker et al., 2018). Before surgery, α-syn PFFs were sonicated in a water-bath cup-horn sonicator for 4 min (QSonica, Q700 sonicator, 50% power, 120 pulses at 1 s ON, 1 s OFF) and maintained at room temperature until injection. Mice 1 month of age were anesthetized with isoflurane. Prior to incision, the animals were injected with local anesthetic into the site of the future wound margin (0.03 ml of 0.5% Ropivacaine; Henry Schein, USA). Mice were then stereotaxically injected bilaterally in the striatum (coordinates from bregma: AP: +0.6 mm; ML: +/− 2.0 mm and DV: −2.6 mm from dura) with 2 μl of PFFs (5 μg/μl); or with matching volumes of vehicle phosphate-buffered saline (PBS) as a control. Injections were made at a rate of 0.4 μl/min using a 10 μl Hamilton syringe with a 34-gauge needle (1.0 in. long, 12-degree beveled tip), mounted on a Stoelting Quintessential Stereotaxic injector (Stoelting). After injection, the needle was left in place for 3 min before being slowly removed. Following surgery, mice received 0.5 ml saline s. c. for hydration and 0.04 ml 0.3 mg/ml Buprenex (Henry Schein) s.c. for analgesia. Injected mice were later anesthetized with sodium pentobarbital (130 mg/kg; Sigma Aldrich) for tissue collection at 2, 4, or 6 months post-intrastriatal injection. Brains were isolated after transcardial perfusion with saline and fixation with 4% PFA. After dissection, brains were post-fixed overnight with 4% PFA and then stored at 4 °C in 30% sucrose in PBS until sectioning. Brains were frozen, and coronal sections of 40 μm were cut on a sliding microtome (Leica, Germany), collected as serial tissue sections spaced by 240 μm, and stored in cryoprotectant.

### 2.3. Immunohistochemical analysis

Brain sections were incubated with primary antibodies for tyrosine hydroxylase (rabbit anti-TH, Millipore, Cat#657012) at a 1:1600 dilution and pS129-α-syn (rabbit anti-pS129, Abcam [51253]) at a 1:1000 dilution. Sections were incubated with respective anti-rabbit biotinylated secondary antisera (Vector Laboratories) and conjugated with an ABC-HRP biotin/avidin complex kit (Vector Laboratories). Staining was developed with 3,3′-diaminobenzidine, and TH-labeled sections were counter-stained with cresyl violet acetate (Sigma-Aldrich). Sections were mounted for imaging and analysis.

### 2.4. Image acquisition

*Z*-stacks of mounted tissue sections processed as above for TH or pSer129-α-syn immunohistochemistry images were captured at 20× magnification using a whole slide scanner (Zeiss, Axioscan Z1) at a 0.22 μm/pixel resolution. Extended depth focus (EDF) was used to collapse the z-stacks into 2D images as they were collected. Tissue thickness was set to 20 μm, z-stacks were collected at 3 μm intervals, and the method setting selected was Contrast. The digitized images were then uploaded to the Aiforia^®^ platform (Aiforia Inc., Cambridge, MA, USA) for analysis with custom deep learning algorithms that were developed in an Aiforia^®^ Create subscription.

### 2.5. Assessment of histology using Aiforia^®^

**Fig. 1.**
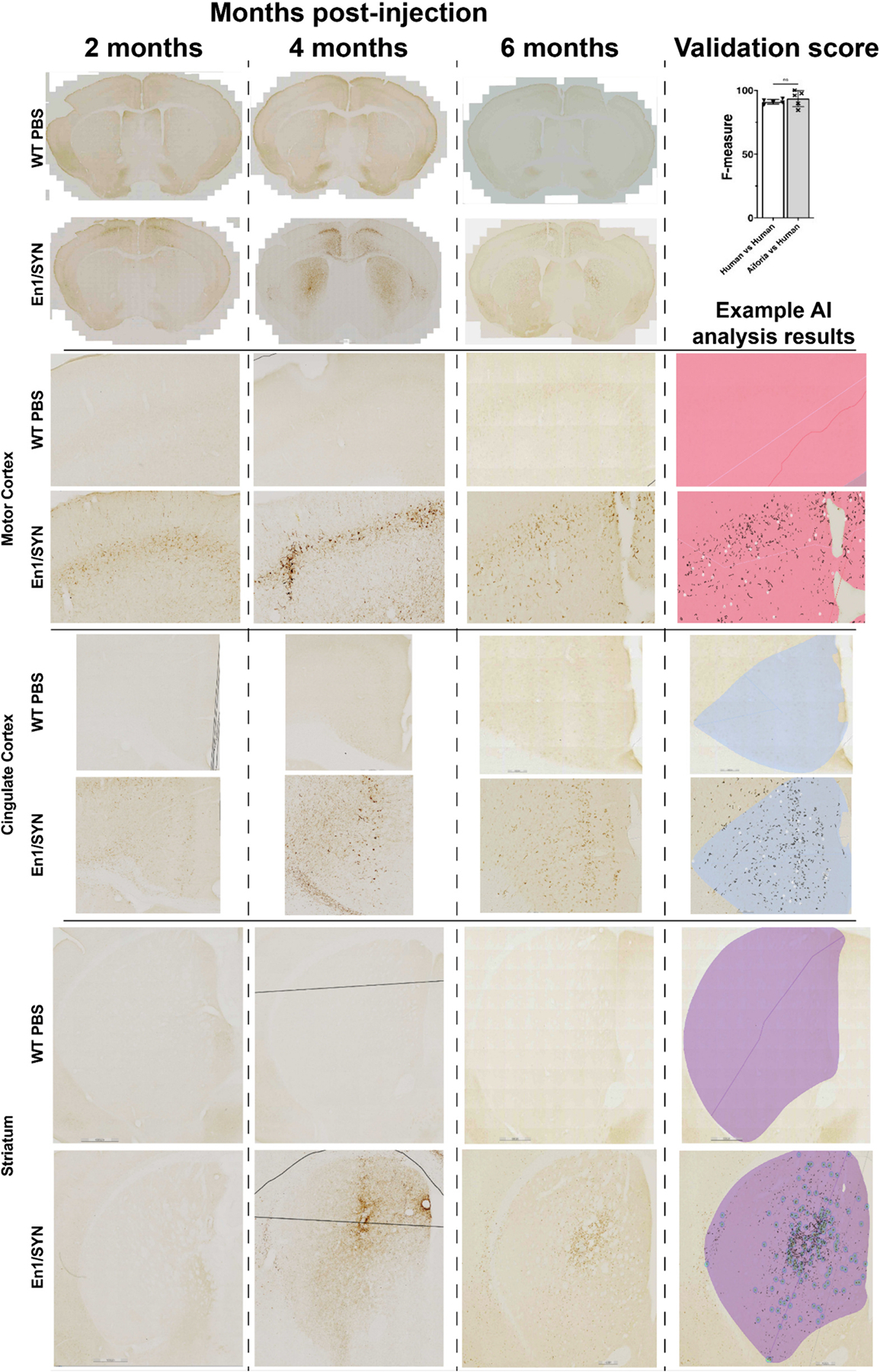

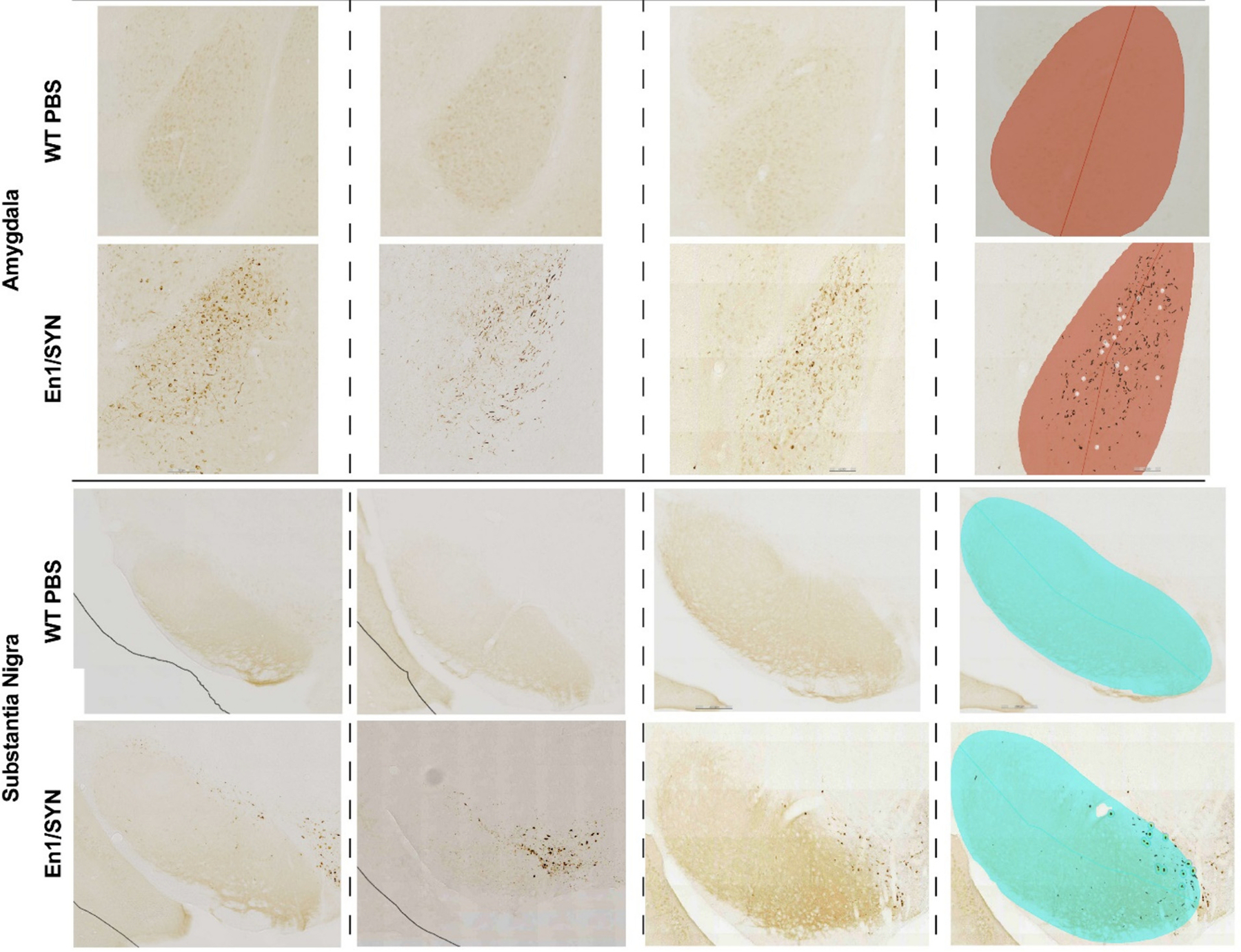
Examples of α-syn pathology and AI model performance between En1/SYN and WT PBS by time point (See data availability for open access to all analysis results): Row 1 Columns 1–3, Coronal cross section examples of α-syn pathology and interobserver variability validation results suggesting no significant difference between Lewy body-like inclusion quantification by AI and researcher annotations (Student’s *t*-test, not significantly different, AI’s F1 = 93.58, SD = 6.33, manual quantification’s F1 = 91.05, SD = 1.81). Rows 2–6 column 1–3 close-up examples of α-syn in specific brain regions. For all AI analysis results in column 4 rows 2–6 α-syn analysis results: black = total pSer129-α-syn, heatmap or white circle = Lewy body-like inclusions, and a brain region specific color mask: pink = Motor cortex, light blue = Cingulate cortex, purple = striatum, red = amygdala.

Supervised deep learning models were trained using scans of coronal mouse brain tissue sections stained for TH or pSer129-α-syn using methodology similar to those previously described (Stetzik et al., 2022). In brief, analysis regions were manually annotated over the right and left hemispheres of each brain section for automated quantification by each model. The TH model was trained to recognize mouse tissue, the substantia nigra (AP −2.6 to −3.9), TH+ cells, and the total area of TH+ immunoreactivity within each cell. It is important to note that this method for TH+ cell quantification in the SN does not include the kind of estimation adjustment features common in unbiased stereology, as a results the total counts are lower. This method has been compared with stereology specifically to provide clarity regarding how to interpret these results (Penttinen et al., 2018). An approximate estimation for adjustment is to multiply the TH+ cell counts reported here by 6 as this was the serial sectioning interval for this tissue. The pSer129-α-syn model was trained to recognize mouse tissue, brain region (striatum, motor cortex, cingulate cortex, amygdala, and SN), total pSer129-α-syn immunoreactivity, Lewy body-like inclusions, and the total area of pSer129-α-syn immunoreactivity within each inclusion. Each AI model was trained on the most diverse and representative images across each respective dataset and validated against blinded human annotations. Whole slide images were loaded to the Aiforia^®^ cloud, and each feature layer was trained using tools in the Aiforia^®^ Cloud platform (see Fig. 1 for examples of pathology and AI model performance).

### 2.6. Behavioral testing

Open field and novel object position recognition (NOPR) behavioral experiments were set up using ANYmaze v.7.2, all behaviors were scored in real-time using video streaming from a Digital USB 2.0 CMOS Camera provided with ANYmaze, and default settings were used for fully automated quantification of animal position and orientation tracking. For both acclimation and testing sessions, animals were placed in the center of the testing apparatus, and behavioral scoring/test-start was initiated manually, in <5 s (approximately) from the time of placement. Once the full testing duration elapsed, scoring stopped automatically. All animals began behavioral acclimation and testing 6 months after intracranial injection of either PFFs or PBS. Animal weights and treatment information were recorded/blinded manually before the start of each test using the protocol and experiment features in ANYmaze v.7.2. The testing schedule varied by experiment type: phenotype validation tests and drug treatment tests (see Table 1).

All behavioral tests were conducted in the small animal behavioral testing suite at the Van Andel Institute (Grand Rapids, MI). Both Open field and NOPR tests used a custom-built flat black 12.7 mm ABS 50 cm × 50 cm × 40 cm testing apparatus for each mouse, constructed in quadruplicate, so each apparatus shared a wall with two other testing environments along adjacent walls, resulting in an apparatus divided into quadrants that was 100 cm × 100 cm × 40 cm. A digital 5 × 5 line grid divided into commonly used Open field zones – sides, corners, middle, and center (exclusive of middle) – was imposed on the testing apparatus floor for zone-specific behavioral spatial metrics.

Animals that were part of the phenotype validation experiments were acclimated to the Open field testing apparatus the day before testing. Both the acclimation and testing durations were 10 min. Following Open field testing, limb coordination was evaluated using a rat-sized rotarod apparatus (San Diego Instruments), with acclimation settings: 4 maximum (max) rpm (day 1) and 16 max rpm (day 2), max duration 300 s, using a linear ramp profile. Testing settings included 40 max rpm, with a max testing duration of 300 s, using a linear ramp profile. Mice were acclimated to the rotarod apparatus across 2 days, running 1 session/day, and were tested in triplicate, one animal at a time. Animals were placed on the rod of the apparatus, runs were started manually, and stopped by an IR fall sensor logging latency and rpm to fall. Gait was assessed using CatWalk XT version 10.6 (Noldus). Animals were acclimated to the walkway across 2 days, running 1 session/day, and were tested until they completed 6 compliant runs. A compliant run was defined as having <60% variability, and a total duration between 0.5 and 30 s. Runs were triggered as an animal entered the camera’s field of view and automatically stopped once the animal exited the camera’s field of view. Percent max speed variability was calculated using default settings in real-time. Before the start of each experiment, a 20 × 10 cm rectangular calibration sheet was used to confirm system calibration (Camera gain 12 dB, green intensity threshold 0.27, green intensity range 0–256).

To determine the response to drugs common in the treatment of movement disorders, Open field locomotor impairment was quantified similarly to the description above but with the inclusion of L-DOPA (l-3,4-dihydroxyphenylalanine) or saline infusion (i.p.) prior to testing. To control for L-DOPA response time variability, the Open field testing period was increased to 2 h (Caudle et al., 2007). To acclimate the animals to a 2-h test, drug treatment test animals were acclimated across 2 days, running 1 session/day before testing. On the day of testing, animals received an injection of either benserazide/L-DOPA (12.5 mg/kg administered 20 min before testing, 15 mg/kg administered immediately before testing) or sterile saline (Fisher Scientific) administered immediately before testing.

Cognitive impairment was quantified using a NOPR test (Cruz-Sanchez et al., 2021; Glasgow et al., 2020). The NOPR test was performed in the OF testing apparatus the day after OF testing, eliminating the need for testing apparatus-specific acclimation. The same 4 painted wooden blocks of similar size but unique from one another in color and shape were used in each test. To control for shared wall effects relative to object-directed orientation or spatial metrics, the orientation and position of the objects were mirrored across all shared walls. Animal orientation was quantified using default “lighter than background” head tracking, and a custom ANYmaze protocol was written to identify each object using both zone (a square extending approximately 20 mm around all sides of an object) and point (object center). NOPR testing was divided into two 10 min testing sessions during which animals could explore the objects. Testing sessions were separated by 10 min during which time animals were returned to their home cages. Prior to the start of the second session, the position of two of the objects was switched. During testing, this object-specific switch was applied to all testing apparatuses running in parallel.

### 2.7. Statistical analysis

Statistical analysis was performed using GraphPad Prism 9 or Rstudio v.1.4.1106. A one-way ANOVA and post hoc multiple comparisons with a Bonferroni correction was used to analyze the following measures: Comparisons of α-syn pathology between timepoint-specific treatment groups were analyzed (number of ROIs by brain regions per animal; striatum 6–8, substantia nigra 4–6, cingulate cortex 4, motor cortex 4, amygdala 2–4), comparisons of α-syn pathology between treatments at the 6-month time points (number of ROIs by brain regions per animal; striatum 6–8, substantia nigra 4–6, cingulate cortex 4, motor cortex 4, amygdala 2–4), all comparisons of TH+ cell counts (number of ROIs in the substantia nigra per animal 8–12). Open field, rotarod, and NOPR locomotor measures. For comparisons of object-directed behaviors in the NOPR, test objects with the same switching status (novel or familiar) were averaged together within a single session by animal and analyzed by an unpaired *t*-test. CatWalk measures were analyzed using an ANCOVA including experimental cohort (*p* = 0.002), testing date (*p* = 0.002), # of runs analyzed (ns), weight (ns), and sex (ns) as co-factors to control for batch effects that were present in the CatWalk results, as it has a higher level of sensitivity compared with the other behavioral measures in this study. AI validation results were also analyzed using an unpaired *t*-test.

AI models were validated using the Aiforia^®^ interobserver variability validation tool to perform a direct validation on the object detectors for the pSer129-α-syn models and the TH+ models (Lewy body-like inclusions and TH+ cells). This “stacked” validation is used to indirectly validate the parent layers (tissue, brain region, and pSer129-α-syn detection) as the performance of the child layers (Lewy body-like inclusions) is dependent on the performance of the parent layers. Using this tool allows for the comparison of inference analysis results with the manual annotations provided by 3 experts in pSer129-α-syn immunoreactivity, in images that did not contribute to the training data. The harmonic mean of the precision and sensitivity values were used to calculate the F1 scores for Lewy body-like inclusions or TH+ cells. F1 scores for “AI versus human” and “human versus human” (interobserver variability) were compared using unpaired *t*-tests, and if the AI’s performance is equal to or better than human annotations then the model is considered valid.

## 3. Results

In this section, we present the results of behavioral tests for motor impairment conducted 6 months after the intrastriatal injection of either PFFs (SYN) or PBS in *En1*+/− or WT mice. We aimed to evaluate motor function in En1/SYN animals injected at 4 weeks of age compared to the WT/PBS group. The Open field and rotarod tests were employed to assess motor function.

### 3.1. Impaired motor function

**Fig. 2.**
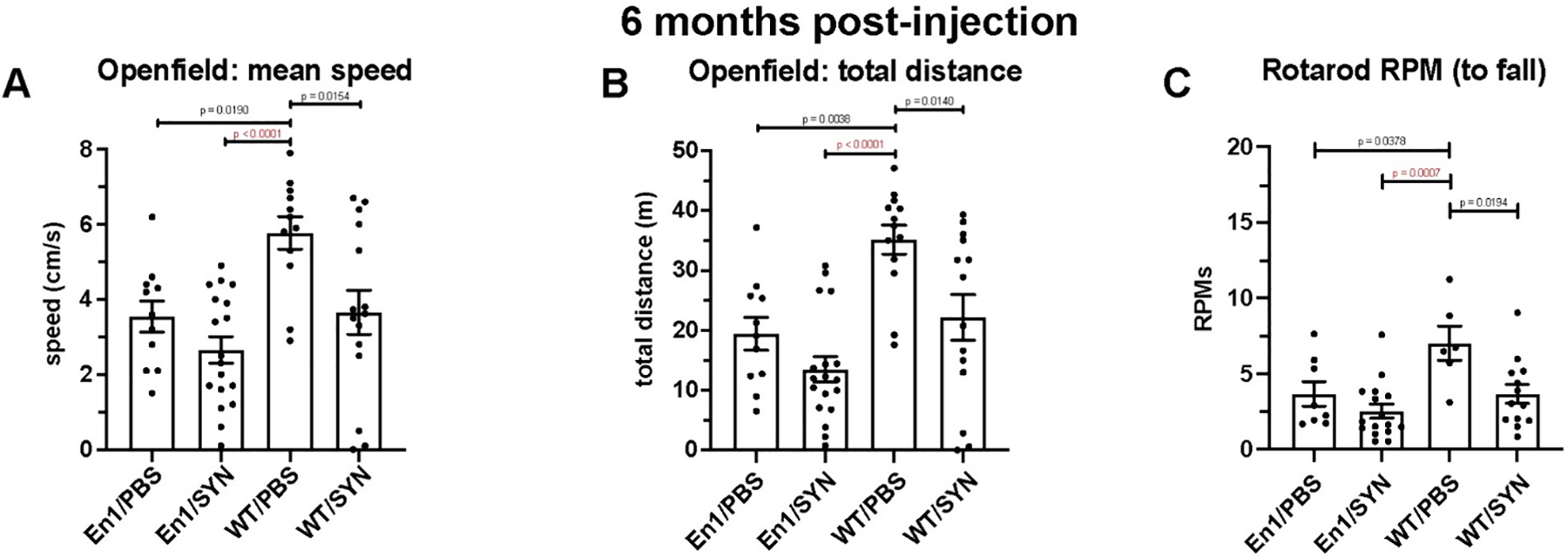
Phenotype validation experiments, part 1 (6 months post-injection; 4 weeks of age): Motor impairment: 1 mouse/marker, bars = treatment group means, error bars (± SEM). One-way ANOVA, multiple comparisons test with Bonferroni correction (see data availability for additional comparisons). *Open field:* En1/PBS *n* = 10, En1/SYN *n* = 17, WT/PBS *n* = 12, WT/SYN *n* = 15. A) Open field mean speed (cm/s): Significant effect of treatment (*p* = 0.0002, En1/SYN vs WT/PBS *p* < 0.0001). B) Open field total distance (m): Significant effect of treatment (*p* < 0.0001, En1/SYN vs WT/PBS *p* < 0.001). *Rotarod Test:* En1/PBS *n* = 7, En1/SYN *n* = 15, WT/PBS *n* = 5, WT/SYN *n* = 13. C) Rotarod RPMs to fall: Significant effect of treatment (*p* = 0.0018, En1/SYN vs WT/PBS *p* = 0.0007).

Our findings from the Open field test indicated a significant reduction in locomotor function in the En1/SYN model compared to the WT/PBS group (mean speed, *p* = 0.019, Fig. 2A; total distance, *p* < 0.0001, Fig. 2B). Additionally, the rotarod test revealed significant limb coordination impairment (RPM to fall, *p* = 0.0007, Fig. 2C).

**Fig. 3.**
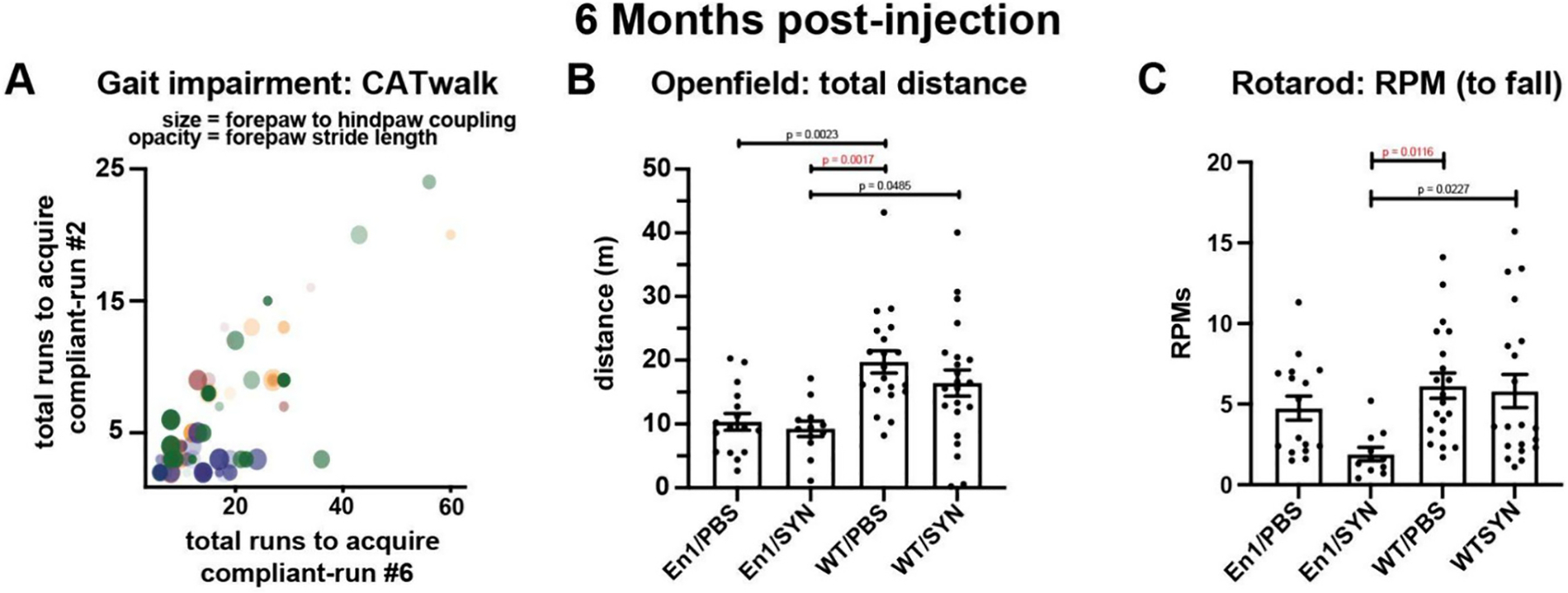
Phenotype validation experiments, part 2 (6 months post-injection; 8 weeks of age): Motor impairment (En1/PBS in yellow, En1/SYN in red, WT/PBS in blue, WT/SYN in green). One-way ANOVA, ANCOVA, multiple comparisons test with Bonferroni correction (see data availability for additional comparisons). A) CatWalk Gait impairment: 1 mouse/marker, En1/PBS *n* = 16, En1/SYN *n* = 12, WT/PBS *n* = 19, WT/SYN *n* = 22. Significant effect of treatment between En1/SYN and WT/PBS on total runs to acquire compliant-run 2 (*p* = 0.002, En1/SYN vs WT/PBS *p* = 0.043), total runs to acquire compliant-run 6 (*p* < 0.001, En1/SYN vs WT/PBS *p* < 0.001), forepaw stride length (*p* = 0.012, En1/SYN vs WT/PBS *p* = 0.045), and left forepaw to right hindpaw coupling (*p* = 0.006, En1/SYN vs WT/PBS *p* = 0.042). B) Open field total distance (m): 1 mouse/marker, bars = treatment group means, error bars (±SEM). Significant effect of treatment (*p* = 0.0003, En1/SYN vs WT/PBS *p* = 0.001). C) Rotarod RPMs to fall: En1/PBS n = 16, En1/SYN *n* = 11, WT/PBS *n* = 20, WT/SYN *n* = 21. Significant effect of treatment (*p* = 0.013, En1/SYN vs WT/PBS *p* = 0.0116).

We then validated the observed loss of motor function in the En1/SYN model, in a group of animals that were injected at 8 weeks of age; at this age En1+/− mice exhibit significant TH+ neuronal loss in the substantia nigra prior to triggering α-syn pathology. We assessed motor impairments using CatWalk, Open field, and rotarod tests. The CatWalk test results revealed a significant treatment effect, emphasizing the robust impact of our interventions. Notably, this effect was prominent enough to significantly influence key parameters. In particular, it influenced the number of total runs required to achieve both the minimum number of compliant runs, specifically 2 runs (En1/SYN vs. WT/PBS, *p* = 0.043), and the maximum number of compliant runs, which are 6 runs (En1/SYN vs. WT/PBS, *p* < 0.001). In CatWalk, each run is “normalized” using automated exclusion criteria such as max speed variability and minimum/maximum run duration. Runs failing to meet these criteria are automatically excluded from the analysis, and while this ensures the consistency for all other comparisons, an increase in number of failed runs could be indicative of motor impairment. Additional CatWalk measures showed significant effects of treatment when comparing the En1/SYN and WT/PBS groups. These measures include left forepaw to right hindpaw coupling (*p* = 0.042) and forepaw stride length (*p* = 0.045, see Fig. 3A).

The Open field and rotarod tests provided further insights into locomotor function 6 months post-injection. These tests collectively suggested a significant loss of locomotor function in the En1/SYN model when compared to the WT/PBS group. Notable outcomes include the total distance covered (*p* = 0.0003, Fig. 3B) and RPM to fall in the rotarod test (*p* = 0.0116, Fig. 3C). These results consistently highlight the impact of our treatment and affirm the observed motor impairments in the En1/SYN model.

**Fig. 4.**
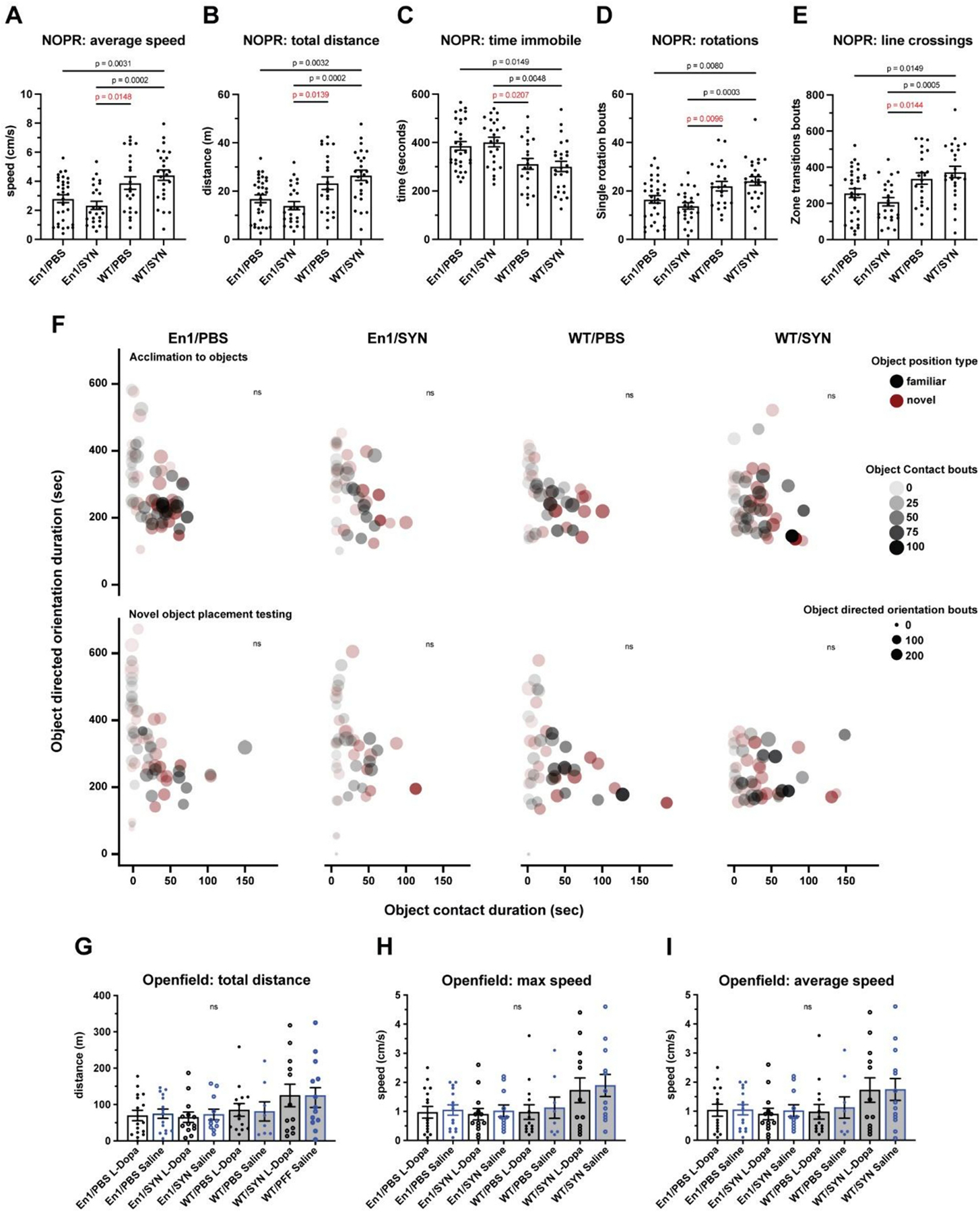
Drug treatment experiments, motor and cognitive impairment, and effects of L-dopa. 1 mouse/marker, bars = treatment group means, error bars (±SEM). One-way ANOVA, multiple comparisons test with Bonferroni correction (see data availability for additional comparisons). NOPR: En1/PBS *n* = 31, En1/SYN *n* = 24, WT/PBS *n* = 22, WT/SYN *n* = 25. A) Average speed (cm/s): (*p* < 0.0001, En1/SYN vs WT/SYN *p* = 0.0002). B) Total distance (m): *p* < 0.0001, En1/SYN vs WT/SYN *p* = 0.0002). C) Time immobile (m): (*p* < 0.0001, En1/SYN vs WT/SYN *p* = 0.0048). D) Rotations (bouts): (*p* = 0.0001, En1/SYN vs WT/SYN *p* = 0.0048). E) Line crossings (bouts): (p = 0.0002, En1/SYN vs WT/SYN *p* = 0.0005). Cognitive Impairment: En1/PBS *n* = 31, En1/SYN *n* = 24, WT/PBS *n* = 22, WT/SYN *n* = 25. F) No significant effects of treatment. 1 mouse/marker = mean values for object-directed orientation vs. object contact, comparing object type (familiar = black, novel = red) during acclimation (top row of bubble plots) and testing (bottom row of bubble plots). Effects of L-Dopa: G–I) Open field: No significant effects of treatment. 1 mouse/marker = mean values for locomotion; total distance (m), max speed and average speed (cm/s). L-Dopa treated (black), Saline treated (Blue), White fill (PFF) black fill (PBS). En1/PBS L-Dopa *n* = 16, En1/PBS Saline *n* = 15, En1/SYN L-Dopa *n* = 13, En1/SYN Saline *n* = 11, WT/PBS L-Dopa *n* = 14, WT/PBS Saline *n* = 8, WT/SYN L-Dopa *n* = 12, WT/SYN Saline *n* = 13.

To determine whether the behavioral deficits observed in the En1/SYN model can be mitigated using a common PD treatment, we conducted experiments on a distinct group of animals. These animals received intrastriatal injections at 8 weeks of age, either with PFFs or PBS, followed by an infusion of either L-DOPA or sterile saline before Open field testing. An in-depth comparison of the treatment groups revealed no significant differences between L-DOPA-treated and saline-treated En1/SYN animals. This suggests that the response to L-DOPA in this context did not result in significant deviations in behavioral performance. To confirm the presence of cognitive impairments in the En1/SYN model, we subjected this group of animals to a novel object placement recognition (NOPR) test. During the testing phase of NOPR, we assessed the duration of time spent in contact with or oriented towards objects with novel placements and compared this with familiar placements. The cognitive evaluation, as measured by the NOPR tests, showed no significant effect of treatment on the duration of time spent in contact with or oriented towards objects with novel placement, in comparison to those with familiar placements. Despite this, a robustly significant treatment effect was observed across all measures of general motor impairments in the NOPR tests (see Fig. 4A–E), exemplified by factors such as average speed (En1/SYN vs. WT/PBS, *p* = 0.0148), total distance (En1/SYN vs. WT/PBS, *p* = 0.0139), time immobile (En1/SYN vs. WT/PBS, *p* = 0.0207), rotations (bouts) (En1/SYN vs. WT/PBS, *p* = 0.0096), and line crossings (bouts) (En1/SYN vs. WT/PBS, *p* = 0.0144). Taken together, these findings illustrate that while L-DOPA treatment did not significantly affect the cognitive aspects of the En1/SYN model, it did influence general motor impairments, suggesting a potential avenue for further investigation and intervention.

### 3.2. PFF-induced α-synucleinopathy

**Fig. 5.**
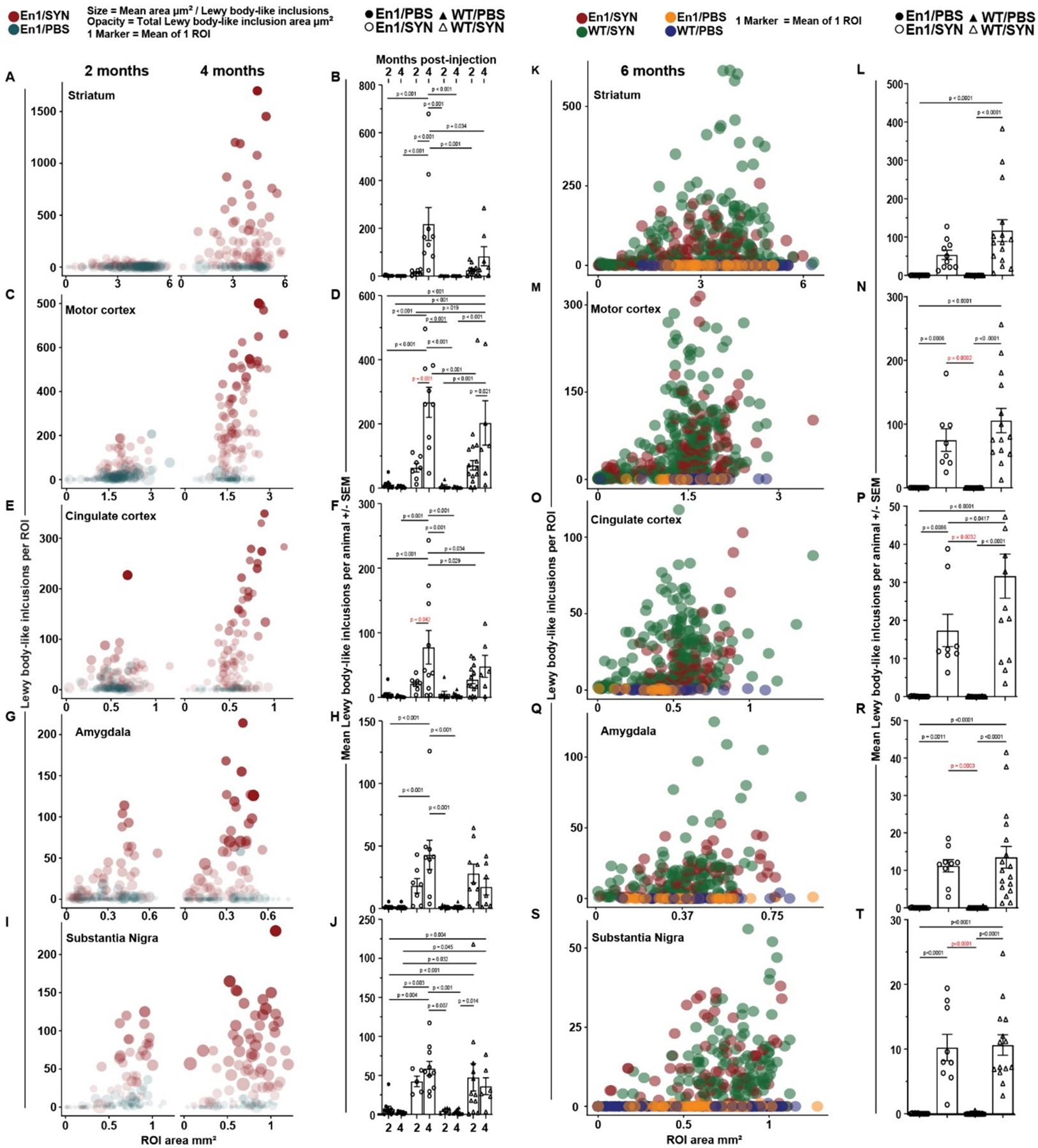
Progressive α-syn pathology (See data availability for open access to all analysis results and additional comparisons). A–J: mice injected with PFFs at 4 weeks of age (2- and 4-months post-injection). Bubble plots - 1 marker/ROI, counts vs ROI area grouped by brain region between post-injection, 2 month (2 m) and 4 month (4 m) time points across injection types, PFF (red markers) and PBS (blue markers), within the En1+/− genotype. Histograms - 1 mouse/marker, bars = treatment group means, error bars ± SEM. 2 m En1/SYN *n* = 7, 4 m En1/SYN *n* = 9. B) Striatum: Significant increases in mean counts of Lewy body-like inclusions between time points within treatment group (*p* < 0.001, En1/SYN vs WT/PBS *p* < 0.001). D) Motor cortex: Significant increases in mean counts of Lewy body-like inclusions between time points within treatment group (*p* < 0.001, En1/SYN vs WT/PBS *p* = 0.001). F) Cingulate cortex: Significant increases in mean counts of Lewy body-like inclusions between time points within treatment group (*p* < 0.001, En1/SYN vs WT/PBS *p* = 0.042). H) Amygdala: No significant phenotype validation relevant increases in mean counts of Lewy body-like inclusions between time points within treatment group (En1/SYN vs WT/PBS *p* > 0.05). J) Substantia Nigra: No significant phenotype validation relevant increases in mean counts of Lewy body-like inclusions between time points within treatment group (En1/SYN vs WT/PBS *p* > 0.05). K–T: mice injected with PFFs at 8 weeks of age (6 months post-injection). Bubble plots - 1 marker/ROI, counts vs ROI area grouped by brain region. Treatment color coding: En1/PBS in orange, En1/SYN = red, WT/PBS = blue, WT/SYN = green. Histograms - 1 mouse/marker, bars = treatment group means, error bars ± SEM. Markers with white fill = PFF and black fill = PBS, circle markers = Engrailed1 and triangle = WT. See data availability for access to all analysis results. Significant increases in mean counts of Lewy body-like inclusions between treatment groups in all observed brain regions. L) Striatum (*p* < 0.0001, En1/SYN vs WT/PBS *p* < 0.0001). N) Motor cortex (*p* < 0.0001, En1/SYN vs WT/PBS *p* = 0.0002). P) Cingulate cortex (*p* < 0.0001, En1/SYN vs WT/PBS *p* = 0.0032). R) Amygdala (*p* < 0.0001, En1/SYN vs WT/PBS *p* = 0.0003). T) Substantia Nigra (*p* < 0.0001, En1/SYN vs WT/PBS *p* = 0.0032).

To determine if the En1/SYN model exhibits progressive development of α-synucleinopathy outside the nigrostriatal system, brains from mice injected with PFFs at 4 weeks of age were collected at 2- or 4-month timepoints and immunostained for pSer129-α-syn. Consistent with our predictions, we observed significant increases in the number of Lewy body-like α-synuclein inclusions at the 4-month timepoint compared to the 2-month timepoint specifically in the motor cortex (*p* = 0.001, Fig. 5C–D) and cingulate cortex (*p* = 0.042, Fig. 5E–F). The results of the pSer129-α-syn analysis suggest that there is a progressive increase in pathological burden that occurs outside the nigrostriatal system, and that this increase is well defined by the increase of Lewy body-like inclusions between the 2- and 4-month timepoints.

At the 6-month timepoint, there were significant differences between En1/SYN and WT/PBS mice in the number of Lewy body-like inclusions outside of the nigrostriatal system (motor cortex *p* = 0.0002 Fig. 5M–N, cingulate cortex *p* = 0.0032 Fig. 5O–P, and amygdala *p* = 0.003 Fig. 5Q–R).

### 3.3. Reduction in nigral TH-positive cells

**Fig. 6.**
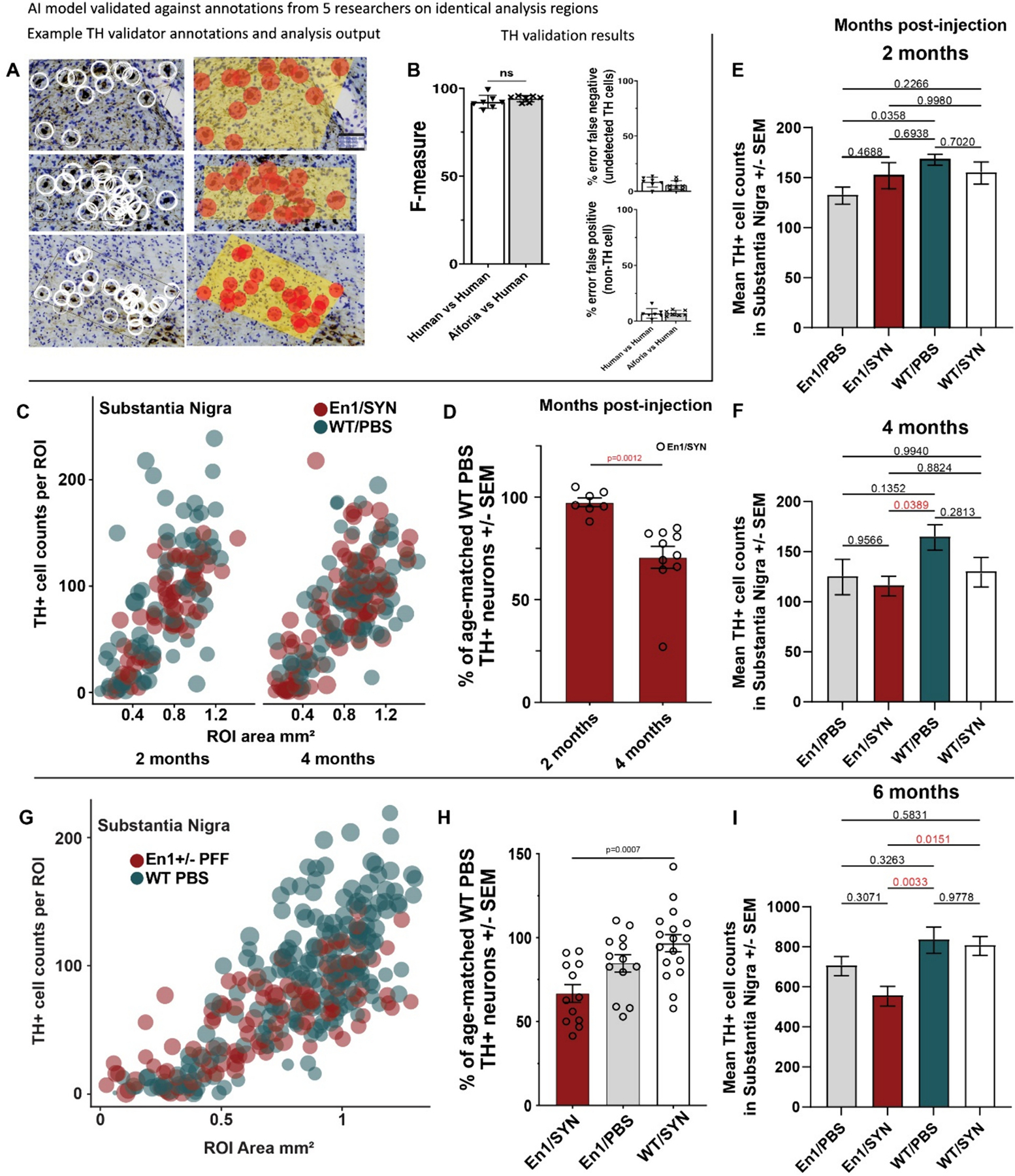
Nigral TH-positive Cell Loss (See data availability for open access to all analysis results and full comparisons). A) Example images of interobserver variability stacked validation, left column annotations from all 5 validators, right column inference analysis results from TH+ cell detection (red markers indicate TH+ cells, and yellow background indicates tissue detection). B) Interobserver variability showing no significant differences between AI performance and consensus from manual annotations from 5 researchers trained in identifying TH+ cells in mice (Student’s *t*-test, not significantly different, Precisio*n* = 92.75%, Sensitivity = 90.14%, False positive error = 7.04%, false negative error = 9.86%, F1 = 91.43). C) Bubble plot - 1 marker/ROI, counts vs substantia nigra ROI area, comparing En1/SYN (red markers) and WT/PBS (blue markers) treatments by time points (2 vs 4 months). D) Histogram - 1 mouse/marker, bars = treatment group means, error bars ± SEM, comparing 2 m En1/SYN *n* = 7 and 4 m En1/SYN *n* = 9 TH+ cell counts as a % of the mean of WT/PBS TH+ cells (Student’s *t*-test, *p* = 0.0012, En1/SYN 2 m mean = 97.45% vs 4 m mean = 70.64%). E) Comparing counts of TH+ cells in the SN across treatment groups at 2 month post-injection (ANOVA, ns). F) Comparing counts of TH+ cells in the SN across treatment groups at 4 month post-injection (ANOVA *p* = 0.0443, multiple comparisons test with Bonferroni correction En1/SYN vs WT/PBS *p* = 0.0389). G) Bubble plot - 1 marker/ROI, counts vs substantia nigra ROI area, comparing En1/SYN (red markers) and WT/PBS (blue markers) treatments at 6 months. H) Histogram - 1 mouse/marker, bars = treatment group means, error bars ± SEM, comparing En1/SYN *n* = 12, En1/PBS *n* = 13, WT/SYN *n* = 17, WT/PBS *n* = 23 mice. TH+ cell counts as a % of the mean of WT/PBS TH+ cells. I) Comparing counts of TH+ cells in the SN across treatment groups at 6 month post-injection (ANOVA *p* = 0.0037, multiple comparisons test with Bonferroni correction En1/SYN vs WT/PBS *p* = 0.0033).

To confirm that En1/SYN mice exhibit a progressive loss of TH-positive cells, TH-positive cell counts in the SN of En1/SYN mice were normalized as a percent of TH-positive cell counts from PBS-injected WT mice from age-matched 2- or 4-month timepoints. Our results show a significant reduction in SN TH-positive cells between 2- and 4-month timepoints (*p* = 0.0012, Fig. 6C–F) as well as a 33.4% reduction at the 6-month timepoint (Fig. 6G–I), indicating a progressive loss of TH+ cells. Additionally, a similar trend of significance is observed in the raw cell counts (Fig. 6E, F & I), however it is important to note that there is no count estimation adjustment calculation applied to the counts as is the convention with unbiased stereology outputs (please see methods, *Assessment of histology using Aiforia^®^* for further clarification).

## 4. Discussion

Our data show that the En1/SYN model exhibits progressive development of α-synucleinopathy accompanied by progressive loss of dopaminergic neurons that together lead to the development of motor deficits. This model has a comprehensive number of PD-like phenotypes that better mimic PD and is therefore superior when compared to the α-syn PFF or En1+/− models alone. The En1+/− model by itself shows many of the features of PD and has been used to develop neuroprotection strategies, and however the En1+/− model by itself does not include α-syn pathology (a key PD feature). The addition of α-syn PFFs to this model increases its usefulness by seeding α-syn pathology.

The interplay between Engrailed-1 (En1) heterozygosity and α-synuclein (α-syn) pathology in the En1/SYN mouse model provides a unique window into the complex mechanisms underlying PD. Engrailed-1 is crucial for the survival of dopaminergic neurons in the substantia nigra, a region severely affected in PD. The heterozygous loss of En1 in our model results in a reduction of mitochondrial complex activity and a progressive degeneration of nigral dopaminergic neurons, which mirrors key aspects of PD pathology. However, this model alone does not replicate the α-synucleinopathy seen in PD.

To address this, we introduced α-syn preformed fibrils (PFFs) into En1 heterozygous mice, thus generating the En1/SYN model. The combination of En1 haploinsufficiency and α-syn pathology creates a ‘dual-hit’ scenario, wherein mitochondrial dysfunction, possibly due to En1 loss, exacerbates the neurotoxic effects of α-syn aggregation. This suggested interaction leads to a non-significant trend in more extensive and progressive development of Lewy body-like inclusions and neurodegeneration beyond the nigrostriatal pathway, affecting multiple brain regions such as the motor cortex, cingulate cortex, and amygdala. The subtle synergistic effect of these two factors — impaired mitochondrial function and α-syn pathology — likely contributes to the significant motor impairments observed in the En1/SYN mice, offering a more comprehensive model of PD.

In this study, we determined that the En1/SYN model displays progressive α-synucleinopathy (Fig. 5), which was expected after the first report of the synergistic effects of inducing α-synucleinopathy in the absence of one En1 allele (Chatterjee et al., 2019). However, the number of Lewy body-like inclusions at 6 months post-injection (Fig. 5) were approximately 30% lower across brain regions than at 4 months post-injection. It is interesting to note that a direct comparison is not possible because the animals quantified at the 4-month time point were injected at 4 weeks of age, while the 6-month animals were injected at 8 weeks of age. While 8 weeks of age is effective in generating the PFFs phenotype and is consistent with the classic methodology for this model (Chatterjee et al., 2019), it is possible that inducing α-syn pathology late in the En1+/− mice (when nigral TH+ neuron loss is already significant) restricts propagation of pathogenic α-syn. Additionally, while quantification of total neuron counts in these regions would be a useful measure to determine if the possible reduction in Lewy body-like inclusions results from neuronal degeneration, total neuron counts are outside of the scope of this study.

The findings from this study underscore the importance of understanding the multi-faceted nature of PD pathology. The En1/SYN model is designed to bridge the gap between mitochondrial dysfunction and synucleinopathy, two central features of PD that have often been studied in isolation. By combining these elements in a single model, with or without synergistic effects, we can better replicate the progressive nature of PD and its impact on both motor and non-motor functions.

Our results show that the En1/SYN model exhibits a broader range of PD-like phenotypes compared to models that focus solely on α-syn pathology or mitochondrial dysfunction. This makes it a valuable tool for studying disease progression and testing potential therapeutic interventions that target multiple pathways involved in PD. Moreover, the lack of significant L-Dopa responsiveness in this model highlights the need for alternative treatment strategies that can address the complex interactions between mitochondrial health and α-syn pathology. The En1/SYN model thus not only provides insights into the disease mechanisms but also serves as a platform for the development of more effective, disease-modifying therapies.

Here, the En1/SYN model demonstrates robust motor impairments across behavioral testing. The locomotor measures recorded during the NOPR test were the most significantly disparate between En1/SYN and control groups. We would like to highlight that the locomotor measures during this test are similar to those recorded during the Open field test with respect to motor impairments. Both Open field and NOPR tests occur in the same testing apparatus, and each stage of the NOPR test is the same duration as the Open field test, thus it is much like running an Open field test in duplicate. Additionally, the number of animals per group for the NOPR tests was nearly double those used in the behavioral validation experiment’s Open field tests. This additional power, as well as running the tests in duplicate, provides the clearest demonstration of the motor impairments in the En1/SYN model. Finally, there was no significant effect of treatment on object-directed orientation or spatial metrics (Fig. 4F). It is interesting to note that the maximum duration of object contact as well as the maximum number of object direct bouts were the highest for the objects with novel placement during NOPR testing, suggesting that some animals possibly recognized the change in object position within the apparatus. Additionally, En1/SYN mice have lower maximal values for these measures than both WT groups. Taken together, the results of the NOPR orientation and spatial metrics suggest that an alternative method, such as a U-water maze, may be more effective in quantifying cognitive impairments.

During the analysis of our CatWalk data, there were clear batch effects related to a number of factors (see methods), possibly masking the effect of phenotype on gait measures. One important consideration relates to the compliance 60% maximum accepted speed variability setting that is consistent with previous methodology in other mouse models (Bernardes and Oliveira, 2017; Zheng et al., 2023). It is possible that a more straightforward characterization of gait impairment could be achieved by modifying run compliance settings to include runs with a variability score as low as 50%, as the impairments in the En1/SYN model were severe enough to exclude many runs in the 50–60% range. This compliant run “filter” is one possible reason that batch effects were more robust in the CatWalk data than in our other behavioral tests.

While the motor impairments of the En1/SYN model are well supported by the behavioral tests in this study, there was no rescue of motor impairment with L-DOPA treatment. It is possible that a different dosing protocol would have been necessary to show responsiveness of the EN1/SYN motor phenotype. The results of the drug treatment experiments suggest that the En1/SYN model is not consistently drug-responsive within treatment groups. There is a trend in the range of values across Open field behavioral measures for L-DOPA-treated mice as being slightly greater than controls, except for the WT/SYN group. This is consistent with the lack of a TH-positive lesion in the SN for WT groups (Fig. 6) and the variability in the number of Lewy body-like inclusions (Fig. 5I–J) in these experiments for the WT groups. While the trends related to our WT groups did not prevent the utility of the En1/SYN model, the design of future studies regarding the background strain could be an important consideration. More broadly, this is consistent with other findings that within populations of PD patients, the response to L-DOPA is also variable (Beckers et al., 2022; Hughes, 1994). This could suggest that the animals that were drug-responsive could be an important focus for future experiments aimed at understanding drug responsiveness (Hwang et al., 2005). However, given the similarity in generic background in En1 mice it is likely that the variability in L-Dopa response is related to variability in Lewy body-like inclusions at a single timepoint. While multiple time points were outside of the scope for this study, a more focused study using only En1/SYN and WT/PBS groups could evaluate a rescue of phenotype at an earlier timepoint for animals that were not responsive at the 6-month time point. One additional factor that could also contribute to the range of L-DOPA responsiveness within groups is the loss of peripheral α-motor neurons associated with the loss of En1 function (Abonce et al., 2019), as L-DOPA is less likely to rescue motor impairments resulting from degeneration of α-motor neurons than due to the loss of midbrain dopamine neurons (Shintaku et al., 2007).

AI models were validated using the Aiforia^®^ interobserver variability validation tool to perform a direct validation on the object detectors for the pSer129-α-syn models and the TH+ models (Lewy body-like inclusions and TH+ cells). This “stacked” validation is used to indirectly validate the parent layers (tissue, brain region, and pSer129-α-syn detection) as the performance of the child layers (Lewy body-like inclusions) is dependent on the performance of the parent layers. Using this tool allows for the comparison of inference analysis results with the manual annotations provided by 3 experts in pSer129-α-syn immunoreactivity, in images that did not contribute to the training data. The harmonic mean of the precision and sensitivity values were used to calculate the F1 scores for Lewy body-like inclusions or TH+ cells. F1 scores for “AI versus human” and “human versus human” (interobserver variability) were compared using unpaired *t*-tests, and if the AI’s performance is equal to or better than human annotations then the model is considered valid. In conclusion, and as expected, our model exhibits both progressive α-synucleinopathy and nigral neurodegeneration that results in motor deficits. Interestingly, and based on the pattern of pathology that we have observed, it is also plausible to expect the development of non-motor deficits as these mice age.

## Figures and Tables

**Table 1:** Behavioral testing schedules.

No pre-testing drug treatment (phenotype validation tests)					
	d1	d2	d3	d4	d5
Open field	Acclimation 10 min	Testing			
Rotarod		Acclimation (4 rpm max)	Acclimation (16 rpm max)	Testing	
CatWalk			Acclimation (2 trials)	Acclimation (3 compliant trials or 10 min)	Testing
Drug treatment tests with L-DOPA or Saline	d1	d2	d3	d4	d5
Open field	Acclimation 10 min	Acclimation 30 min	Testing		
Novel object placement recognition				Testing (OF apparatus, no acclimation needed)	

## Data Availability

Extracted data will be made available on request, analysis results can be viewed using the links provided: Phenotype validation experiments part 1&2, TH+ Cell Loss: https://cloud.aiforia.com/Public/VANANDEL_BrundinLab/lmI8LhIXa4wUNncr9zPLxdsghRk1pERguSTg0-sMIUI0 Phenotype validation experiments part 1&2, progressive α-syn pathology:https://cloud.aiforia.com/Public/VANANDEL_BrundinLab/oUlDW_kxDBxIJW0xdKli1dZhLGrdlM9s4v3LkIqrWBU0
